# Weightlifting Overhead Pressing Derivatives: A Review of the Literature

**DOI:** 10.1007/s40279-019-01096-8

**Published:** 2019-03-28

**Authors:** Marcos A. Soriano, Timothy J. Suchomel, Paul Comfort

**Affiliations:** 1 Department of Physical Activity and Sport Sciences, University of Murcia, San Javier, Spain; 2 Directorate of Sport, Exercise and Physiotherapy, University of Salford, Frederick Road Campus, Statham St, Salford, Manchester, M6 6NY UK; 3 Department of Human Movement Sciences, Carroll University, Waukesha, WI USA

## Abstract

This review examines the literature on weightlifting overhead pressing derivatives (WOPDs) and provides information regarding historical, technical, kinetic and kinematic mechanisms as well as potential benefits and guidelines to implement the use of WOPDs as training tools for sports populations. Only 13 articles were found in a search of electronic databases, which was employed to gather empirical evidence to provide an insight into the kinetic and kinematic mechanisms underpinning WOPDs. Practitioners may implement WOPDs such as push press, push jerk or split jerk from the back as well as the front rack position to provide an adequate stimulus to improve not only weightlifting performance but also sports performance as: (1) the use of WOPDs is an additional strategy to improve weightlifting performance; (2) WOPDs require the ability to develop high forces rapidly by an impulsive triple extension of the hips, knees and ankles, which is mechanically similar to many sporting tasks; (3) WOPDs may be beneficial for enhancing power development and maximal strength in the sport population; and, finally, (4) WOPDs may provide a variation in training stimulus for the sports population due to the technical demands, need for balance and coordination. The potential benefits highlighted in the literature provide a justification for the implementation of WOPDs in sports training. However, there is a lack of information regarding the longitudinal training effects that may result from implementing WOPDs.

## Key Points

**Table:** 

This review provides information regarding historical, technical, kinetic and kinematic mechanisms, and potential benefits and guidelines to implement WOPDs as training tools for the sports population.
Strength and conditioning coaches may implement WOPDs such as push press, push jerk or split jerk to improve not only weightlifting performance, but also to enhance sports performance.
WOPDs may provide a variation in training stimulus for the sports population due to the technical demands, need for motor control and coordination, and the ability required to develop force rapidly through a closed kinetic chain.

## Introduction

In 1925 the Fédération Internationale Haltérophile (FIH) published the first authentic list of World Records including the following exercises: one-hand (right and left) snatch, one-hand (right and left) clean and jerk (C&J), two-hand press, snatch and C&J [1–3]. Introduced at the Amsterdam Olympic Games in 1928, the weightlifting program was limited to three main lifts: the two-hand press, the snatch and the C&J [1, 4]. However, these three lifts lasted until 1972, when the press was omitted from official competitions making way for the modern era of weightlifting, which is composed of the snatch and C&J movements (for more information, see https://www.iwf.net/weightlifting_/history/ [1, 3, 4]). Nonetheless, the historical background presented above shows that weightlifting overhead pressing derivatives (WOPDs) have been a big part of the weightlifting competition for an extended period.

The study of weightlifting exercises and their derivatives has been of great interest to researchers and strength and conditioning coaches [5–9]. More recently, the underpinning biomechanical characteristics of such exercises have received notable attention [10–21] to assist in more effective programming of such exercises [21–23].

The snatch and C&J are complex whole-body movements performed using a series of high-intensity muscular actions. Weightlifters are required to generate high peak forces, rates of force development and impulse in order to adequately accelerate the barbell to lift more than their opponents, consequently resulting in high power outputs [24–26]. The importance of weightlifting movements and their derivatives to train lower body muscular power for optimising the force-velocity profile of athletes [23, 27] as well as for enhancing performance in different sporting tasks such as vertical and horizontal jumps [28–32], sprinting and change of direction [31, 32] has been extensively investigated and reported. Furthermore, previous research findings support that weightlifting exercises and their derivatives may train an athlete’s ability to ‘absorb’ a load during impact activities [13, 23, 33], which, hypothetically, might be important for training deceleration [33].

Weightlifting movements may be further subdivided into weightlifting catching, pulling and pressing derivatives [34–36]. Weightlifting catching derivatives require athletes to perform the catch phase; however, in the case of the power clean or power snatch the bar is not caught in a full squat position. The catching derivatives include the following: power clean and power snatch from various positions including the floor, hang at knee, and hang at thigh. Additionally, these lifts can be performed from blocks/plinths at the knee and thigh. In contrast, weightlifting pulling derivatives are those where the catch phase is excluded. Examples of weightlifting pulling derivatives include the snatch and clean pulls from the floor, knee or thigh. These can be performed from a hang or blocks/plinths. The jump shrug, the high pull or the hang high pull are also derivatives that fall into this category [21, 23, 27, 37–40]. Weightlifting derivatives have been used extensively over the history of weightlifting [41–47]. Despite the fact that weightlifting exercises and their pulling and catching derivatives have been well studied [21, 23, 27], little is known about the group of overhead pressing derivatives.

The jerk is not technically a pressing motion; rather the athlete accelerates the bar vertically via extension of the hips, knees and ankles, while dropping underneath the bar into the catch position. The jerk has been shown to be the exercise in which the greatest weight is lifted overhead in weightlifting competitions [24, 26, 48]. Supporting evidence also indicates that this exercise is excellent for achieving high levels of power output and improving muscular power in athletes [24, 49, 50]. Moreover, the jerk and other WOPDs such as push press, push jerk or split jerk from the back are widely implemented in strength and conditioning programs [51–54], based on the notion that they are mechanically similar to many sporting skills, due to the rapid extension of hips, knees and ankles [55].

The aim of this review was to present empirical evidence to provide an insight into the kinetic and kinematic mechanisms underpinning WOPDs. We focused on not only the weightlifting performance but also their application to resistance training programs to enhance sports performance.

## History of Overhead Pressing Exercises in Weightlifting

Since the origins of weightlifting, overhead pressing derivatives have played a large part in the history of this sport. When the press was omitted from competitions, there was a change in methods of application that were reported in the literature [41]; however, WOPDs were still being implemented and recommended by practitioners [42]. It is of interest to consider two clear stages in the history of weightlifting: before the abolition of the press and after the abolition of the press.

### Weightlifting: Before the Abolition of the Press

Not so long ago, the press was considered a gold standard by which the strength capability of an athlete was measured [1, 2, 4, 56–58]. In fact, when weightlifting competitions and rules became standardized at the Amsterdam Olympics in 1928, the clean and press was adopted as a true measure of overall strength, along with the ‘quick lifts’, i.e. the C&J and the snatch [3, 4]. For 50 years the clean and press was included as part of the international weightlifting program. Eventually, the International Weightlifting Federation (IWF) decided at the meeting celebrated in 1972 in Munich to abolish the press from all future competitions.

**Fig. 1:**
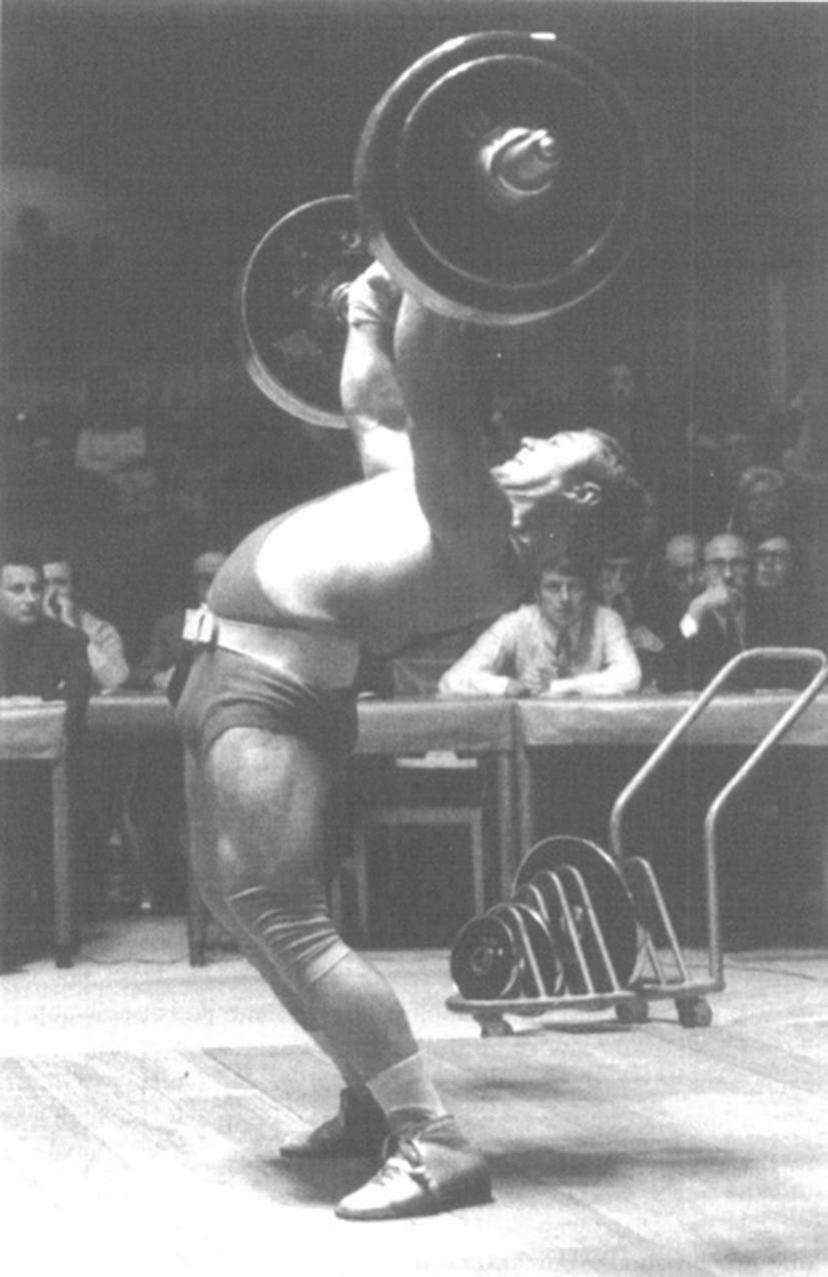
The continental press. A picture of Valerij Yakubovsky at the international meet in Brussels, Belgium in 1971. With permission and courtesy of Dr John D. Fair [4]

The word ‘press’ in weightlifting was associated with lifts where the barbell was raised in a slow and steady motion, using predominantly the strength of the arms [56, 58]. However, it was not the technique seen in the following decades [1, 4, 57, 59]. The press performed slowly and steadily proved to be impractical for lifting heavy loads, and various ways of ‘cheating’ were developed, enabling lifters to use the larger muscle groups of the legs, hips and lower torso, instead of relying on just upper-body muscles, which resulted in the famous style known as the ‘continental press’ [1, 3, 4, 57, 59]. A graphic representation of the continental press can be seen in Fig. 1—a considerable quick backbend before the lift characterised it, which enabled the lifters to drop the trunk under the bar, resulting in higher loads lifted overhead [1, 3, 4, 59].

Eventually, the disparities in judging the press technique became greater for several reasons, for example politics, supremacy, the need to win by any means, etc., and along with a long list of lower back injuries due to the accentuated backbend drove the IWF to eliminate the press from all future competitions starting from the following year [1, 3, 4, 41]. Nonetheless, it is worth noting the incredible feat that the renowned Vasily Alekseyev achieved with a world record and the highest score ever seen at Tallinn National Championships (Soviet Union), pressing 236.5 kg, which now makes up part of the weightlifting history annals.

In the ‘old’ era of weightlifting, competitors who had poor results in the snatch and C&J could improve their total by performing a good press. As such, Laputin and Oleshko [41] observed large improvements in the press in comparison to the snatch and the C&J in the results of the last 20 years of the ‘old’ weightlifting era (1952–1972).

Previously, weightlifters trained more on the press and its assistance exercises, resulting in a decrease in the number of assistance exercises for the snatch and the C&J. In fact, Roman [43] showed that the volume of training previous to the abolition of the press was comprised of: 30% pressing exercises, 22% snatch, 16% C&J, 17% squats, 13% snatch and clean pulls, and 2% other complementary exercises.

### Weightlifting: After the Abolition of the Press

In the old era of weightlifting, lifters could attain good results or even a win by performing a strong press; however, in modern weightlifting (after the abolition of the press) success is determined by the quick and powerful lifts (snatch and C&J). Therefore, modern coaches and lifters are required to develop more strength-speed abilities as well as technical proficiency [41, 42, 60]. There was a general need by coaches and lifters to change methodologies and training philosophies, and these changes had as a main goal to preserve or even to increase the previous results, but in the area of modern weightlifting [41, 43, 61].

Training load was not decreased—rather it was invested to increase scores in the snatch and C&J. These main exercises, as well as pulling and catching derivatives, were increased, while pressing exercises were undertaken to a lesser degree [41]. Classic pressing exercises were replaced by other exercises, such as the push press, push jerk and other jerk derivatives in an attempt to perfect the jerk technique [42, 43, 60]. In fact, Roman [43] suggested the following exercise ratio for modern weightlifting: 27% snatch, 26% C&J, press 10%, squat 20%, pulls 15% and 2% other exercises. However, this generated certain controversies that have been addressed in the literature, and some authors have suggested a lesser training volume for pressing exercises around 10% [36, 43], while others proposed greater emphasis of around 20% [62]. Nonetheless, current literature and weightlifting manuals [47, 51, 53, 54, 63–65] still suggest including WOPDs for improving technique, overall motor coordination and power development, not only for weightlifters but also for general preparation in athletes.

## Previous Literature on Weightlifting Overhead Pressing Derivatives

Previous literature focused on the technique of the different WOPDs: standing press [53, 64, 66, 67], push press [53, 68–70] and jerk [45, 53, 60, 71]. Additionally, much of the weightlifting information focusing on the exercise technique is found in different weightlifting manuals [1, 36, 41, 43, 51, 54, 63].

Little research has been conducted to date regarding the kinematic and kinetic variables of WOPDs [16, 24, 26, 50, 72, 73]. Garhammer [24, 26] reported the occurrence of great power outputs in the jerk thrust (the propulsive phase where the lifter pushes the bar vertically by an impulsive lower body triple extension to reach the overhead position) of up to 6952 W developed by top male and female weightlifters. However, recent studies reported power output values for the jerk of around 3100 W [16, 74]. These differences may be due to Garhammer [24, 26] evaluating world class weightlifters and due to the different devices, loads and methodologies used to assess power output, resulting in significantly different perceived power outputs [75–77]. Additionally, recent research reports high power outputs (3000–5600 W) in the push press exercise [78–80], similar to values reported for exercises with similar lower-limb kinematics, such as the jump squat or power clean [19, 75, 80].

Although WOPDs have been a big part of weightlifting and sport training history, there is a gap between practitioners and the body of scientific knowledge. Therefore, deeper and more detailed research is needed to provide information regarding the kinetic and kinematic mechanisms underpinning WOPDs, as well as potential adaptations to training.

## Literature Search Methodology

A search of electronic databases was conducted to identify all publications on *weightlifting overhead pressing derivatives* up to May 2018. The literature search was undertaken using 15 different keywords: ‘overhead exercises’, ‘pressing exercises’, ‘weightlifting’, ‘biomechanics’, ‘kinematics’, ‘kinetics’, ‘jerk’, ‘split jerk’, ‘clean & jerk’, ‘overhead press’, ‘military press’, ‘Olympic press’, ‘standing press’, ‘push press’, ‘push jerk’. Search terms were combined by Boolean logic (AND, OR), with no restrictions on date or language, in PubMed, Medline (EBSCO) and Google Scholar databases. We also extended the search spectrum to ‘related articles’ and the bibliographies of all retrieved studies.

### Inclusion Criteria

The following inclusion criteria were used to select articles focused on WOPDs and the biomechanical analysis of the studies:

I. Full-text, research articles exploring and analysing any WOPDs were selected. As such, case studies, review articles, and articles that did not present research were excluded.
II. Research articles must have reported insight into either kinetics or kinematics of the exercise/s analysed.

Articles that met the inclusion criteria were additionally classified by temporality to show descriptively the progression developed in this field to date, and also by type of exercises to provide an insight in the exercises studied to date.

### Methodological Quality of Included Studies

Study quality was evaluated by a standard procedure (see Table 1). Each study was read and ranked from 0 to 6, with the larger number indicating better quality. For each question, a 1 was awarded if the study met the standard. If insufficient description or data were provided to analyse a specific question, a 0 was awarded. The score was then tallied for each question, with the highest score possible equalling 6 out 6. The evaluation process was conducted by two researchers (initial evaluators) who ranked the articles blinded. Then, a third researcher (mediator) compared the scores of each researcher. If there was a consensus on the scores, the score remained, but, if there was no consensus, the three researchers involved (initial evaluators and mediator) discussed the study to provide the definitive score.

**Table 1:** Criteria list for the methodological quality assessment

No.	Item	Score
1	Sample description: + Properties of the subjects (age, weight, height, sex) + Definition of the population (well-trained, recreationally trained or untrained) + Training status and training years in strength or power training	0 or 1
2	Procedure description: + Detailed description of the test (exercise and loading conditions employed) + Detailed description of the intervention protocol (randomised order to exercises, developed exercises in different days and order for all subjects)	0 or 1
3	Intervention: + Defined and supervised exercises technique (bar position, depth of the half-squat, elbows extension) + Defined number of trials to lifts + Defined adequate recovery between trials across all lifts	0 or 1
4	Instruments and methods employed for kinetic and kinematic calculation: + A FP method and the combined method (FP + 3D motion) employed in the assessment was valued as quality criteria for ballistic exercises (PP, PJ and SJ), since the ground reaction forces measured or calculated using a FP provide the most accurate method to assess forces during lower-body ballistic exercises [81, 82]. Furthermore, both methods have shown an agreement in measuring power output during ballistic exercises [83]. Moreover, when an LPT, accelerometer or any other kinematic device was employed assessing the velocity of the bar, only bar mass should have been used for power output calculations. However, when an FP was employed, both the bar mass and the lifter’s mass (system of mass) should have been used following the guidelines provided by Hori et al. [84]	0 or 1
5	Measurement system, data collection, and data analysis: Instrument description (brand, model and origin country of the product) + Defined sampling frequency + Defined configuration and variable calculation of the instrument + Defined and developed reliability test when proceed + Defined collection software for recording and analysing data	0 or 1
6	Results detailed + Measure of the central tendency + Variation or dispersion from the average	0 or 1

*FP* force platform, *3D* three-dimensional, *PP* push press, *PJ* push jerk, *SJ* split jerk, *LPT* lineal position transducer

## Results

### Study Characteristics

**Fig. 2:**
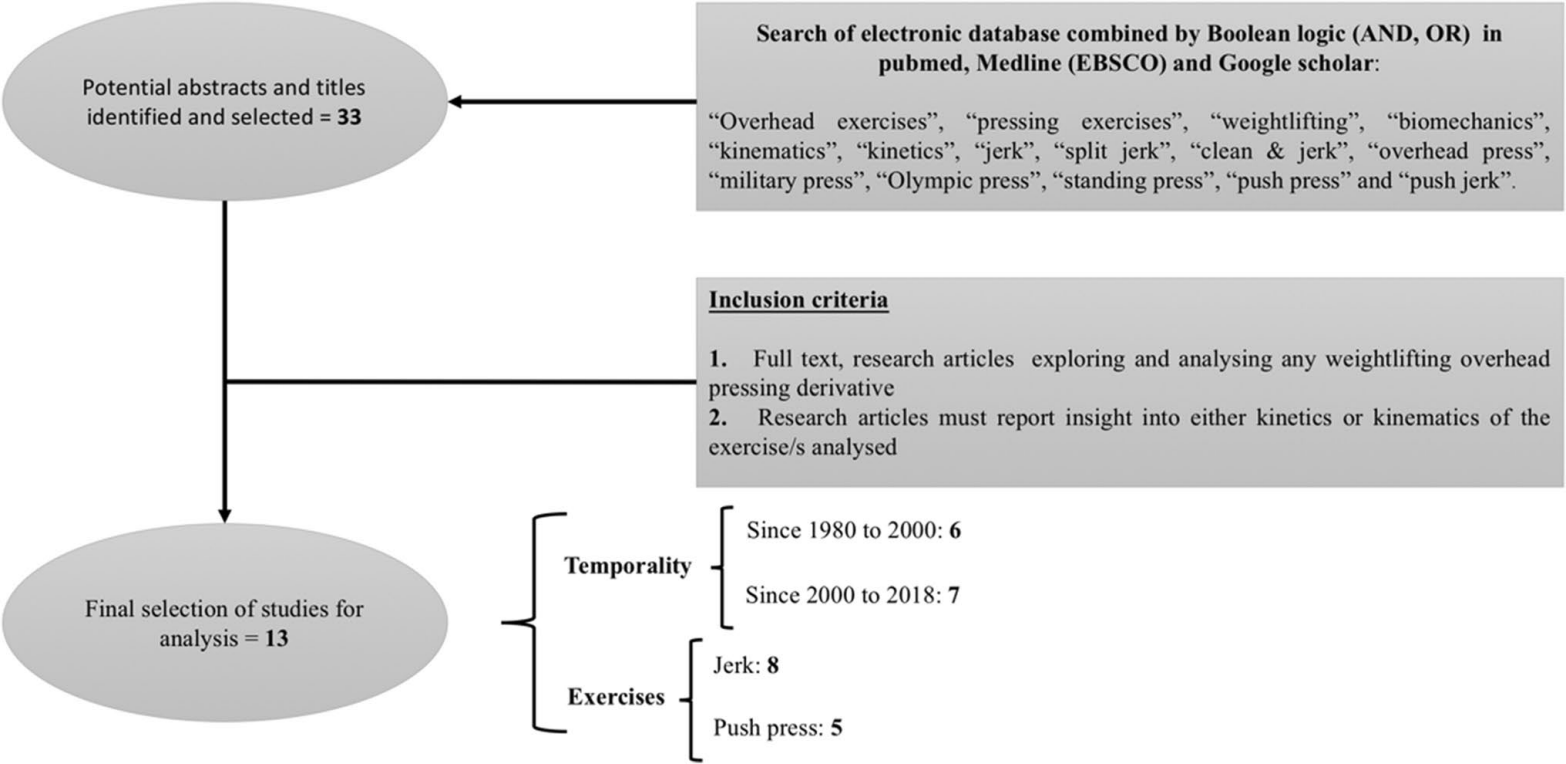
Flow diagram of the study selection process and description of the final selection

A flow diagram of the literature search and the final selection is shown in Fig. 2. According to the above-defined inclusion criteria, we identified 13 independent studies [16, 24, 26, 50, 72–74, 78–80, 85–87]. An overview of the main information from these studies can be found in the following sections, where the WOPDs and variations (see Table 2) and the main information regarding kinetics and kinematics results of the different studies analysed is provided (see Table 3) to guarantee a deeper knowledge of the WOPDs. Quality scores ranged from 3 to 6 points: 3 points—23.1%, 5 points—30.8%, and 6 points—46.1%.

**Table 2:** Characteristics of the main weightlifting overhead pressing exercises and classification of the complementary variations

	Standing press (SP)	Push press (PP)	Jerk (J)
Nature of the exercise	Non-ballistic exercise	Ballistic exercise	Ballistic exercise
Main muscles actively employed	Upper body muscles (shoulder girdle and deltoids)	Flexors and extensors of the lower body and upper body muscles (shoulder girdle and deltoids)	Flexors and extensors of the lower body and upper body muscles through the trunk for stabilization
Description of the contraction	Pure upper-body maximal concentric contraction	Impulsive triple extension (ankles, knees and hips) characterized by a SSC of the lower body transmitted through the trunk to an upper body concentric contraction	Impulsive triple extension (ankles, knees and hips) characterized by a SSC of the lower body transmitted through the trunk and a posterior knee rebending to catch the bar under in the overhead position. The trunk, lower and upper body are working to find balance and stabilization once the lifter is under the bar
The position of the barbell	SP from the chest (military press) SP from the back (behind the neck)	PP from the chest PP from the back (behind the neck)	J from the chest J from the back (behind the neck)
The hand spacing	SP (clean grip) SP (narrow grip) SP (snatch grip)	PP (clean grip) PP (narrow grip) PP (snatch grip)	J (clean grip) J (snatch grip)
The drop under the barbell			Split J (Scissors) Push J Squat J

*SP* standing press, *PP* push press, *J* jerk, *SSC* stretch shortening-cycle

**Table 3:** Descriptive characteristics and kinetic and kinematic results of different studies conducted on WOPDs

Study	Sample characteristics	Training status	Methods	Results	Quality score
Lake et al. [74]	*n* = 7 subjects Age: ND Sex: B Height: 174 ± 4 cm BM: 81.5 ± 14.6 kg	Recreational weightlifters 1RM C&J: ND ND about experience	Exercise reviewed: J Loads employed: 80% 1RM C&J (65 ± 20 kg) Assessment devices: 2 FP (200 Hz) High-speed video camera (200 Hz)	1. The PRFD results were: 17.2 ± 4.86 BW·s^−1^ 2. The estimated relative PPO values were: 34 ± 9.5 W·kg^−1^ and absolute PPO 3046 ± 472.5 W 3. The dip phase duration was 460 ± 0.08 ms	5
Grabe et al. [50]	*n* = 27 subjects G1 (Master) = 5 Sex: M Age: 18 years (17–20 years) Height: 167 cm (162–173 cm) BM: 629 N (587–662 N) G2 (the rest of WL classes) = 22 Age: 16.8 years Sex: B Height: 163.5 cm (150–174 cm) BM: 578 N (387–662 N)	G1 Professional weightlifters (master) 1RM C&J: ND > 2 years S-P experience G2 Recreational weightlifters 1RM C&J: ND > 2 years S-P experience	Exercise reviewed: J Loads employed: 100% 1RM J (maximum attempts) Assessment devices: High-speed video camera (30 f·s^−1^)	1. Master lifters were characterized by a shorter braking phase of 0.136 ms and a greater range of trunk inclination during the split) 2. The dip phase duration was 0.226 ms 3. The depth of the dip was shallower 12.3 % than any other group, correlated with the duration of the braking phase (*r* = 0.65; *p* < 0.01). 4. Master athletes presented the best ratio in maximum ascending/descending velocities: 1.23 m·s^−1^ respect to the rest of the groups 5. The peak ascending velocities (PV) in the thrust were in this study 1.16 m·s^−1^ less than the optimal range for lightweights (1.4 m·s^−1^) regarding the importance of the height and weight in developing maximum ascending velocities 6. The duration of the split was 0.285 ms and was also similar to that of elite athletes in other studies	5
Häkkinen et al. [72]	*n* = 13 subjects G1 = 7 subjects Age: 24.9 ± 3.6 years Sex: M Height: 165.3 ± 6.9 cm BM: 76.0 ± 17.3 kg G2 = 6 subjects Age: 26.5 ± 5.5 years Sex: M Height: 172.7 ± 7.1 cm BM: 76.3 ± 13.2 kg	G1 Professional weightlifters (elite) 1RM C&J: 147.9 ± 29. 7 kg > 2 years S-P experience G2 Recreational weightlifters (district) 1RM C&J: 114.3 ± 25.3 kg > 2 years S-P experience	Exercise reviewed: J Loads employed: 70, 80, 90, and 100% 1RM C&J Assessment devices: FP Electrical goniometer High speed video camera (40 f·s^−1^)	1. The PGRF during the J thrust decreased with the increased of barbell load (*p* < 0.05) in both groups. However, the elite group showed a plateau for loads of 70 and 80% 1RM C&J (210%) and it decreased for the following loads until 188%. The district group followed an almost linear pattern from 198 to 168%. Nonetheless, elite group showed higher PGRF across the spectrum of loads 2. The AV of the barbell during the J thrust decreased significantly (1.5–1.1 m·s^−1^, *p* < 0.05) in the elite group as the load of the barbell increased. However, for the district group there was a different pattern, peak bar velocity increased from 1.15 to 1.18 m·s^−1^ from loads of 70–80% and then decreased to 1.05 m·s^−1^ following the same pattern as the elite group. Nonetheless, elite group showed higher barbell velocities, specially for loads of 70 and 80% 1RM C&J 3. The average knee angular velocity during the J thrust decreased as the load increased for elite and district groups (from 238 to 218 and from 258 to 242 rad s^−1^). The average knee angular velocities were similar for both groups 4. The mean time values of the DUB increased as the load of the barbell increased from 128 to 150 ms for the elite group and from 160 to 178 ms for the district group. The elite group was significantly faster at every load level as compared to the district group (*p* < 0.01–0.05)	6
Garhammer [24]	*n* = 8 subjects Age: ND Sex: M Height: ND BM: 87.8 kg (different categories from 52 to 142 kg)	Professional weightlifters (elite) 1RM C&J: ND ND about experience	Exercise reviewed: J Loads employed: 100% 1RM C&J (maximum attempts) Assessment devices: High-speed video cameras (50 f·s^−1^)	1. PPO in the J was on average 3491 W (2503–4786 W) depending on the lifter’s BM. Although there were exceptions, there was an increase in PPO with BM 2. Descriptively, the J thrust had similar power values than the 2nd pull of clean and snatch	3
Kauhanen [73]	*n* = 13 subjects G1 = 7 subjects Age: 24.9 ± 3.6 years Sex: M Height: 165.3 ± 6.9 cm BM: 76.0 ± 17.3 kg G2 = 6 subjects Age: 26.5 ± 5.5 years Sex: M Height: 172.7 ± 7.1 cm BM: 76.3 ± 13.2 kg	G1 Professional weightlifters (elite) 1RM C&J: 147.9 ± 29.7 kg > 2 years S-P experience G2 Recreational weightlifters (district) 1RM C&J: 114.3 ± 25.3 kg > 2 years S-P experience	Exercise reviewed: J Loads employed: 70–100% (10% increase) of 1RM C&J Assessment devices: FP Electrical goniometer High speed video camera (40 f·s^−1^)	1. A significant difference (*p* < 0.05) was observed in the duration of the DUB (split) of the J for the elite group in comparison to the district group (149.3 vs. 178 ms, respectively). It was also supported by a negative correlation between the relative J results and the duration of the drop under the bar (*p* < 0.05) 2. All the time parameters such as duration of the preparatory dip and J thrust were shorter than for the district group (preparatory dip: 487 vs. 555.3 ms; J thrust: 258.5 vs. 276.2 ms; respectively), although not statistically significant 3. All the forces exerted either: eccentric max. force or concentric max. force were greater for the elite group than for the district group (ecc forces: 178.3 vs. 164.3%; con forces: 185.7 vs. 170.7%), although not statistically significant 4. The PV of the barbell was faster for all the parameters assessed (preparatory dip, J thrust and drop under the bar) in the elite group than in the district group (dip: − 0.44 vs. − 43 m·s^−1^; thrust: 1.11 vs. 1.06 m·s^−1^; drop: 2.48 vs. 2.15 m·s^−1^), although not statistically significant 5. Maximal con forces were correlated positively with knee angles at the eccentric–concentric coupling phase (lesser the knee flexion, greater the force) (*p* < 0.01) and negatively with the duration of the eccentric phase (*p* < 0.001)	6
Lake et al. [78]	*n* = 17 subjects Age: 25.4 ± 7.4 years Sex: M Height: 183 ± 5 cm BM: 87 ± 15.6 kg	Recreationally trained PP 1 RM: 78 ± 13 kg ND about S-P experience	Exercise reviewed: PP Loads employed: 10, 20, 30, 40, 50, 60, 70, 80 and 90% 1RM PP Assessment devices: FP (500 Hz)	1. PPO was maximized at 75% 1RM (3200 W), whereas MPO was maximized at 65% 1RM (2050 W) 2. JS PPO was 6.7% greater than PP; whereas PP MPO was 10.3% greater than JS MPO 3. The impulse applied during PP with the OL for PPO was lower in PP than in the JS (247.8 vs. 278.7 N·s, respectively). However, there were no difference between MPO for both exercises (233.9 vs. 256.9 N·s, respectively) 4. PP training with the OL could provide a stimulus sufficient to elicit a lower-body power training response 5. PP impulse was maximized with the heaviest load due to the time available to apply force is constrained in this exercise	5
Comfort et al. [80]	*n* = 11 subjects Age: 22.2 ± 3.5 years Sex: M Height: 176.5 ± 5.56 cm BM: 85.78 ± 14.29 kg	Recreationally trained 1RM PP: 85.4 ± 8.3 kg > 2 years of S-P experience	Exercise reviewed: PP Loads employed: 50, 60 and 70% 1RM PP Assessment devices: 2 FP (1000 Hz)	1. PP PPO varies across loads (50% 1RM: 3676 ± 1020.3 W, 60% 1RM: 4071.1 ± 1552.3 W, 70% 1RM: 1976.2 ± 1416 W), although it was not statistically significant with other exercises and loads (SJ: 50% 1RM: 4257.5 ± 1081.1 W, 60% 1RM: 4430.4 ± 1140.3 W, 70% 1RM: 4195.4 ± 1212 W; MTPC: 50% 1RM: 4479.3 ± 1357.2 W, 60% 1RM: 4352.5 ± 1319.6 W, 70% 1RM: 4739.2 ± 1015.8 W) 2. All the exercises (PP, SJ and MDPC) and load conditions (50, 60, and 70% 1RM) may be used interchangeably without any detrimental effect on PPO when focusing on improving power development	6
Comfort et al. [85]	*n* = 11 subjects Age: 23 ± 3.5 years Sex: M Height: 178.6 ± 8.5 cm BM: 88.7 ± 13.5 kg	Recreationally trained 1RM PC: 98.9 ± 8.59 kg > 2 years of S-P experience	Exercise reviewed: PP Loads employed: 60% 1RM PC Assessment devices: FP (1000 Hz)	1. PF in the PP (2607 ± 435 N) was not statistically significant in comparison to SJ (2795 ± 522 N) and MDPC (2928 ± 302 N), although MDPC resulted in the highest PF 2. PRFD in the PP (13,959 ± 6821 N·s^−1^) was not statistically significant in comparison to SJ (11,998 ± 4885 N·s^−1^) and MDPC (14,243 ± 4216 N·s^−1^), although MDPC resulted in the highest PRFD 3. PPO in the PP (3708 ± 956 W) was not statistically significant in comparison to SJ (4052 ± 605 W) and (3810 ± 636 W), although SJ resulted in the highest PPO 4. All the exercises may result in similar adaptive responses when focusing on improving rate of force development (strength speed) in athletes	6
Loturco et al. [87]	*n* = 27 subjects Age: 18.4 ± 1.2 years Sex: M Height: 178 ± 0.7 cm BM: 74.4 ± 9.5 kg	Elite soccer players 1RM PP: ND ND about S-P experience	Exercise reviewed: PP Loads employed: from 30% BM up to decrease in MPP Assessment devices: LPT (1000 Hz)	1. MPV was higher in the PP than JS (1.65 ± 0.22 vs. 1.04 ± 0.09 m·s^−1^, respectively) 2. MPP was higher in PP than in JS (727 ± 134.8 vs. 698 ± 113.1 W, respectively) 3. JS was more related to lower-limb neuromechanical abilities in team-sport athletes (soccer players) than PP	5
Garhammer [26]	*n* = 5 subjects Age: ND Sex: M Height: ND BM: 89.6 kg (different categories from 55.7 to 138.5 kg)	Professional weightlifters (elite) 1RM C&J: 198.8 kg (from 147 to 240 kg) ND about experience	Exercise reviewed: J Loads employed: 100% 1RM C&J (maximum attempts) Assessment devices: High-speed video cameras (50 f·s^−1^)	1. The average of these subjects was 5184 W for PPO (3548–6953 W) and 3734 W for MPO (2825–4321 W) during the J thrust 2. The average barbell velocity was 1.74 m·s^−1^ for the J thrust (from 1.6 to 1.9 m·s^−1^) 3. The efficiency value was 99% during the J thrust. It means that the percent of total work done in the lift resulted in vertical as opposed to horizontal motion 4. Maximum velocities and PPO during the J were closely related to those during the snatch and clean	3
Garhammer [86]	*n* = 9 subjects Age: ND Sex: F Height: ND BM: 62.4 kg (different categories from 43.9 to 82.6 kg)	Professional weightlifters (elite) 1RM C&J: ND ND about experience	Exercise reviewed: J Loads employed: 100% 1RM C&J (maximum attempts) Assessment devices: High speed cameras (100 Hz)	1. PPO for women in the J thrust is very similar in magnitude (42.5 W·kg^−1^; 1866–3510 W) to the women’s average relative power output values for snatch and clean 2nd pulls 2. Power output values for the J have been shown to compare closely in magnitude to those for snatch and clean 2nd pulls	3
Flores et al. [16]	*n* = 13 subjects Age: 25.9 ± 6.9 years Sex: M Height: 174.7 ± 3.3 cm BM: 72.2 ± 9.9 kg	Well-trained weightlifters 1RM J: ND 1RM J from the back: ND > 2 years of S-P experience	Exercise reviewed: J and J from the back Loads employed: 30, 40, 50, 60, 70, 80 and 90% of 1RM J. 30, 40, 50, 60, 70, 80 and 90% of 1RM J from the back Assessment devices: Accelerometer (100 Hz)	1. The J and J from the back PPO increased from 30 to 90% 1RM. Furthermore, the J from the back elicited a greater PPO than the J for all the loads assessed 2. PPO occurred at a relative intensity of 90% 1RM for the J (3103.34 ± 616.87 W) and the J from the back (3400.22 ± 691.07 W). However, these results were not significantly different from the peak power produced with 80% for both exercises	6
Winwood et al. [79]	*n* = 6 subjects Age: 24 ± 3.9 years Sex: M Height: 181.6 ± 28.9 cm Weight: 112.9 ± 28.9 kg	Well-trained strongman athletes 1RM C&J: 116.7 ± 20.4 kg > 2 years of S-P experience	Exercise: PJ/PP and LL Loads employed: 70% 1RM C&J Assessment devices: FP High speed cameras (1000 Hz)	1. PPO (5629 ± 1565 W) and MPO (2960 ± 802 W) in the PJ/PP were lower than PPO (6629 ± 2068 W) and MPO (3831 ± 1079 W) during the 2nd pull phase in the clean 2. PPO (5629 ± 1565 W) and MPO in the PJ/PP (2960 ± 802 W) were higher than PPO (3527 ± 1172 W) and MPO (1758 ± 586 W) during the 1st pull phase in the clean and the log lift PPO (3699 ± 618 W) and MPO (1922 ± 591 W) 3. PV (1.82 ± 0.09 m·s^−1^) and MV (0.97 ± 0.08 m·s^−1^) in the PJ/PP were lower than PV (2.18 ± 0.17 m·s^−1^) and MV (1.69 ± 0.15 m·s^−1^) during the 2nd pull phase in the clean 4. PV (1.82 ± 0.09 m·s^−1^) and MV in the PJ/PP (0.97 ± 0.08 m·s^−1^) were higher than PV (1.51 ± 0.2 m·s^−1^) and MV (0.75 ± 0.15 m·s^−1^) during the 1st pull phase in the clean and also the log lift PV (1.6 ± 0.1 m·s^−1^) and MV (0.88 ± 0.07 m·s^−1^) 5. Impulse in the PJ/PP (345.6 ± 66.8 N·s) was greater than any other part of the C&J exercise (1st pull: 291.8 ± 95.2 N·s; 2nd pull: 164.7 ± 88 N·s) and also the log lift (306.9 ± 56.8 N·s)	6

*M* men, *F* female, *B* both (male and female), *BM* body mass, *WL* weightlifting, *S-P strength-power*, *1RM* one repetition maximum, *ND* no data, *G1* group 1, *G2* group 2, *C&J* clean & jerk, *J* jerk, *PP* push press, *PJ* push jerk, *MDPC* mid-thigh power clean, *SJ* squat jump, *JS* jump squat, *CMJ* counter-movement jump, *PC* power clean, *FP* force platform, *LPT* lineal position transducer, *PPO* peak power output, *PRFD* peak rate of force development, *GRF* ground reaction force, *PV* peak velocity, *MV* mean velocity, *DUB* drop under the bar, *MPO* mean power output, *OL* optimal load, *PF* peak force, *MPP* mean propulsive power, *MPV* mean propulsive velocity

### Weightlifting Overhead Pressing Derivatives: Description, Variations and Main Kinetics and Kinematics Mechanisms

The characteristics of the main overhead pressing derivatives are presented in Table 2. These include the nature of the exercise, muscle actions and primary muscles actively employed [47, 51, 53]. Additionally, the position of the barbell, hand spacing and drop under the bar in the jerk may be subdivided into these main exercises into different complementary exercises such as standing press from the back, snatch grip push press or push jerk [47, 53, 88].

#### The Standing Press

The standing press is a complex, multi-joint movement that mainly involves the upper body muscles to lift the load, although the trunk and the lower body provide stability for the development of the lift. The technique of the standing press has been well described elsewhere [64, 66, 67]. The standing press has been extensively used in strength training and rehabilitation programs [53, 89, 90].

To our knowledge there are no data on power development during standing press to date, although this may be due to its common use for strength and hypertrophy rather than power development. There is just one study that analysed the kinematics of the bar, where the mean propulsive velocity (MPV) was measured through an incremental loading test [91]. However, the test was performed in a Smith machine and subjects were seated on a bench, which contributed to decreasing the role of the trunk and lower body for stabilization [70]. Additionally, this study used a linear position transducer (LPT) to assess barbell velocities, which may impact the results, resulting in higher velocities and power compared to calculations from force-time data collected from a force platform [92, 93]. It is well documented that the use of barbell velocities using an LPT or other kinematic device for power calculations results in greater power outputs when compared to calculations based on velocity of the centre of mass (COM) calculated from force-time data using a force platform [19, 75, 81, 94].

Moreover, kinematic and kinetic variables of the standing press may be hypothetically variable according to the different complementary forms employed (from the chest vs. behind the neck, snatch grip vs. clean grip, etc.) as was shown in one study during the jerk [16]. Eventually, the standing press seems to be more applicable to sports performance than the well-known bench press due to the development of force through a close kinetic chain [69, 70]. Consequently, we suggest that more studies should be developed to understand the biomechanical mechanisms underpinning pressing performance.

#### The Push Press

The push press has been well described by O’Shea [69, 70], and the main characteristics and variations are detailed and summarised in Table 2. It is a complex, powerful multi-joint exercise that generates large forces by the muscles of the lower body, transmitting these through the trunk to the upper extremities, which is the main difference with respect to the standing press [53, 68–70]. The use of the lower body includes two key movements known as the dip (unweighting and braking phase of a quick partial squat) and the thrust or drive (a very rapid propulsion phase via extension of the hips and knees and plantar flexion of the ankles). These phases are also presented in the different variations of the jerk; they are related to weightlifting and other sporting tasks such as jumping, and are considered crucial for developing high power outputs [41, 49].

Kinetics, kinematics and power development during the push press have been investigated by different authors as summarised in Table 3. Lake et al. [78] found that the peak power output during the push press was not significantly different compared to the jump squat (3,640.1 ± 573.8 vs. 3,885.2 ± 302.3 W, respectively). However, mean power output in the push press was significantly greater than jump squat mean power (2313.6 ± 332.5 vs. 2096 ± 201.8 W, respectively). Furthermore, although the loads at which peak and mean power were maximized tended to be larger in the push press, there were no significant differences during the jump squat [peak power: 81.3 ± 9.9 vs. 52.5 ± 25.5% one-repetition maximum (1RM); mean power: 63.8 ± 16.9 vs. 38.8 ± 34% 1RM, respectively].

In a recent study, Winwood et al. [79] conducted a biomechanical analysis of overhead pressing exercises in which they allowed either the push jerk or the push press to be performed, but they did not differentiate the data for the two exercises—instead, they put them all together. The authors found high peak and mean velocities (1.82 ± 0.09 and 0.97 ± 0.08 m.s^−1^, respectively) and peak and mean power outputs (5629 ± 1565 and 2960 ± 802 W, respectively) exhibited by the push press/jerk lifts. The power values were slightly higher than those reported by Lake et al. [78], likely due to the fact that Winwood et al. [79] used a fixed load that corresponded to the 70% 1RM of the C&J, whereas Lake et al. [78] used the 1RM of the push press. In addition, there were also differences in training status between the subjects employed in the two studies.

Loturco et al. [87] conducted a study in which they found a higher MPV and mean propulsive power (MPP) in the push press compared to the jump squat (MPV: 1.65 ± 0.02 vs. 1.04 ± 0.09 m.s^−1^; MPP: 727 + 134.8 vs. 698 + 113.1 W, respectively), although the jump squat was more related to sprinting and jumping abilities tested in elite soccer players than the push press [87, 95]. However, Loturco et al. [87, 95] employed a LPT to assess barbell velocity and subsequently calculate power output, which limits the comparison to studies where a force platform was used to collect force-time data and subsequently calculate system velocity and power [81, 96].

Although power development in the push press exercise has been studied, there is a lack of information on the kinematic and kinetic variations related to the different complementary exercises.

#### The Jerk

The jerk has been well described in different manuals and studies published in the literature [43, 45, 51, 53, 71, 97]. In addition to the main characteristics of the jerk, which are described in Table 2, the jerk is a unique exercise where the largest loads are lifted to an overhead position. Furthermore, it is the only sporting undertaking in which a human being has been able to lift three times their body mass overhead [48].

Such incredible attributes led to some studies focusing on the main kinematic and kinetic characteristics of the jerk and the differences between successful and unsuccessful lifts and master lifters and less experienced lifters [50, 73, 97, 98]. Current evidence (Table 3) suggests that in order to develop a successful lift, the half-squat, the thrust and the drop under the bar are the key variables to the jerk [24, 26, 97, 98].

The dip phase (half-squat) of the jerk involves three crucial phases, similar to the dip during the counter-movement jump (CMJ). These phases consist of the quick dip (unweighing phase), the braking phase (deceleration at the bottom of the dip) and the propulsion (thrusting) phase [98]. A strictly vertical movement and optimal time-duration and displacement during a half-squat have been shown to be the key difference between master and novice lifters [24, 26, 43, 50, 72, 73, 86, 97–99]. A slower half-squat may decrease force potentiation in the subsequent propulsion phase by a reduction in muscle spindle stimulation and elastic energy potentiation [50, 97, 98], although a longer duration increases the time in which force can be applied, which may result in a greater net impulse and therefore greater acceleration. In contrast, a very quick half-squat may decrease the subsequent impulse needed to accelerate the bar overhead as a result of a decreased net impulse because of the reduced duration [50, 97, 98]. The thrust propulsion phase shows the highest bar speed values (from 1.4 to 1.8 m·s^−1^) and, consequently, the highest power outputs [24, 26, 43, 49, 50, 72, 73, 86, 97–99].

The last phase of the lift is the drop under the bar, where the athlete lowers his/her centre of mass (COM), catching the bar in the overhead position [45, 60, 98, 100]. The most common styles are the split, the push and the power style. It is important to note that although power and push jerk are commonly used interchangeably refer to different styles. In the push jerk, also referred to as the power jerk, the feet remain in contact with the platform rather than being lifted and replaced. However, we use the push jerk to refer to both terms in this review. Additionally, there is a challenging technique known as the squat jerk [48, 63, 88] (see Table 2). The squat jerk is predominantly used by Chinese lifters, but the snatch balance is widely used by weightlifters worldwide as an assistance exercise to train the receiving phase (catch) of the snatch. The snatch balance is mechanically similar to the squat jerk exercise, and just the hand spacing is changed to decrease the barbell height needed to complete the lift, consequently providing a greater challenge to the mobility and stability of the lifter [44, 101]. Although kinetic and kinematic differences between jerk styles have not been widely studied to date, the split jerk is the preferred style for weightlifters [48, 51, 88]. However, every lifter has his/her own individual peculiarities, and a more in-depth study of the differences in the kinematic and technical parameters of the jerk and their variations would be necessary to provide accurate information to strength and conditioning (S&C) coaches in order to prescribe effective training methods to improve not only the jerk performance in experienced weightlifters, but also sports performance for athletes and practitioners.

Studies that have analysed the kinetic parameters of the jerk performance have shown that not only are the greatest loads lifted to an overhead position, but also very high values of power outputs have been developed [24, 26, 49, 86]. As such, Garhammer [24, 26, 86] found very high values from 2500 to 6953 W for peak power and 2690–4321 W for mean power during the jerk. In contrast, other studies have shown lower values than those cited above: Lake et al. [74] and Flores et al. [16] found values of 3046 W and 3103 W, respectively, for the peak power during the jerk.

The differences found in the studies summarised in Table 3 should be based on the fact that power development is influenced by the training status of the sample employed [73, 102]. The method of assessment and subsequent calculation of velocity and power are likely the most influential factors in the differences in values reported. Most of the studies presented in Table 3 have assessed barbell velocities [16, 24, 26, 50, 86, 87]. During weightlifting the bar and system of mass (bar + body mass) do not move in parallel, thus the use of barbell velocities determined via displacement-time data, collected via LPT or video, for power calculations results in greater velocities and therefore power outputs when compared to calculations based on velocities of the system COM calculated from force-time data using a force platform [19, 75, 81, 94]. However, although different studies have been conducted on the validation and assessment of the lower body kinetic performance during weightlifting exercises such as power clean, hang power clean or mid-thigh pull [17–19, 48, 94, 96], little is known about which is the most accurate methodology to obtain valid power output values in the overhead pressing exercises and, most specifically, in the jerk.

Additionally, characteristics such as the position of the barbell and the drop under the bar are said to change the power output values as mentioned above. Flores et al. [16] conducted a study on the differences between the jerk from the back versus the jerk from the chest across different relative intensities. The main findings were that the jerk from the back elicited greater power output than the jerk from the chest for all the loads assessed, although the peak power output occurred at a relative intensity of 90% of 1RM for both exercises and was greater, but not significantly, for the jerk from the back than the jerk from the chest (3400.22 ± 691.07 and 3103.34 ± 616.87 W, respectively).

## Potential Benefits of Weightlifting Overhead Pressing Derivatives

As mentioned above, WOPDs may be seen as useful activities for improving weightlifting performance [41, 42, 51], motor control and coordination [25, 70, 103] and achieving high levels of power development that may enhance performance not only in experienced weightlifters but also for the general sport population [24, 26, 78, 88, 104].

### Weightlifting Performance

The jerk from the chest has been presented as one of the most complex and difficult skills in the modern era of weightlifting. In fact, the jerk is the part of the clean and jerk that shows the highest incidence of failure in weightlifters [48, 97, 98, 105]. It is a reciprocal process where not only the complexity of the movement is a critical factor, but also the great amount of load lifted overhead, which increases its technical demands.

For instance, Ivanov and Roman [98] described in the *Russian Weightlifting Yearbook* how 20% of weightlifters at the 1980 USSR National Championships were disqualified from competition due to their inability to fix the barbell at the jerk portion. Similarly, Herrera [105] developed a study that collected results of snatch and C&J attempts in competitions over a period of 6 years (1972–1977) for Cuban weightlifters. The results showed that the mean number of snatch records exceeded that of the C&J and that the main cause of failure was in the jerk portion, about 60%. Thus, the findings confirmed that Cuban weightlifters of all age groups, even some record holders, primarily commit errors in the jerk. Nonetheless, a deeper knowledge of the jerk and also a greater amount of time devoted to it would be very beneficial in improving the jerk technique and, consequently, weightlifting performance.

Different strategies have been suggested to manage the jerk technique and also to improve weightlifting performance [41, 43, 97, 98]. On the one hand, there are the use of special-assistance exercises such as the jerk from the back, the jerk from stands or blocks, snatch grip jerk or half-jerk, for targeting and setting efficacy in the different phases of the jerk technique [51, 63, 97]. On the other hand, barbell velocity values for the thrust phase are approximately 0.2 ± 0.25 m·s^−1^ lower than those observed when the press was still part of the weightlifting competitions [41, 43, 98]. Considering that the weightlifters used to have a greater volume of WOPDs at that time [41], this could have improved the overall upper body strength and power levels of weightlifters. All in all, the results suggest that in order to more successfully execute the jerk, it could be useful to increase the use of WOPDs in order to achieve greater levels of strength, barbell velocities and, consequently, power output.

Finally, practical applications may be suggested based on the studies cited above. Weightlifters still have considerable problems with the jerk, and besides some strategies suggested to address the missed attempts, these occurrences show the necessity to study the biomechanics and mechanisms underpinning the jerk.

### Motor Control and Coordination

O’Shea [70] and a more recent review by Bishop et al. [68] have emphasised the importance of WOPDs and especially the push press as an alternative for strength and conditioning programmes. WOPDs derivatives require the ability to develop force through the kinetic chain from the lower to the upper extremities, which may be a powerful stimulus to strengthen muscles of the upper and lower body while optimising motor control and coordination, due to the key role of the trunk and lower body muscles in stabilising and transmitting forces in a closed kinetic chain [53, 90, 103, 106]. Specifically, the push press was compared with the well-studied bench press, suggesting that WOPDs such as push press or jerk and variations are more applicable to explosive events and sports than the bench press due to the technical challenges requiring speed, acceleration, timing and coordination [68–70].

### Enhancing Power Development in Sports

WOPDs may be a powerful tool for enhancing sport performance in a wide range of sport populations mainly for two reasons: (1) WOPDs develop high levels of maximal strength and power; and (2) WOPDs are mechanically similar to many sporting tasks.

#### Weightlifting Overhead Pressing Derivatives Develop High Levels of Maximal Strength and Power Development

Current evidence shows that implementing weightlifting training may be a good stimulus to develop rapid force production, maximal strength and power in a sporting population [23, 55, 88, 104, 107–109]. Specifically, evidence suggests that weightlifting training enhances athletic performance that requires high-load speed strength [109]. According to Hori et al. [109], the jerk is the exercise where the largest loads are lifted to an overhead position, and, furthermore, to succeed in the lift, it has to be performed as quickly as possible [48, 50, 86, 99]. The combination of the two variables, the force, due to the heavy loads that can be lifted, and velocity, due to the high barbell speeds, result in a complete and perfect stimulus to achieve high levels of power output that may target the ability to develop rapid force production and also power development necessary to enhance athletic performance [49, 88, 109].

Some studies found similarities in kinetic and kinematic variables between WOPDs and other weightlifting and ballistic exercises [78, 80, 85]. On the one hand, Lake et al. [78] concluded that the mechanical demand of the push press is comparable to that of the jump squat, enabling the lifter to apply significantly greater power over the propulsion phase of the triple extension (hips, knees and ankles) with less mechanical cost than the jump squat. As a result, the push press could provide an effective stimulus and a time-efficient combination to target the entire kinetic chain during strength and power training. On the other hand, Comfort et al. [80] suggested that when training to maximise peak power output and also rapid force production, the mid-thigh power clean, squat jump and push press performed at 50–70% 1RM could be used interchangeably without detriments in power development. Moreover, Comfort et al. [85] reported no differences in peak force, peak power or rate of force development (RFD) during the mid-thigh power clean, squat jump and push press using 60% 1RM power clean, suggesting that any of these exercises could be used to target rapid force and power production under moderate loads.

Finally, Garhammer [49] reported in a review of power output studies of weightlifting and powerlifting exercises that the jerk thrust, together with the snatch and clean second pulls, are the exercises where the highest levels of power output are achieved. Consequently, the results presented suggest that WOPDs may be an adequate stimulus when training to maximize power and rapid force development. However, more research should be conducted to assess the biomechanical parameters of WOPDs and the optimal loads to develop maximal power production.

#### Weightlifting Overhead Pressing Derivatives are Mechanically Similar to Many Sporting Tasks

Weightlifting exercises and derivatives are believed to enhance sport performance due to the rapid extension of the hips, knees and ankles that occurs in many sporting activities [55]. Moreover, weightlifting training has been effective at improving performance in other sporting activities such as sprinting, jumps and change of direction [31, 32]. In fact, weightlifting training causes different adaptations in the knee muscle co-activation in comparison with traditional resistance training, and may result in a superior enhancement of sport performance [110].

More specifically, some studies have compared the similarities between WOPDs and specific sporting activities [87, 95, 111–113]. Cushion et al. [111] compared the loaded push jerk and jump squat and a countermovement jump (CMJ). Unexpectedly, the push jerk was more related mechanically to the CMJ than the jump squat, which is one of the exercises commonly used to improve jumping abilities. Additionally, evidence suggests that the jerk appears to offer an effective strategy to overload joint moment generation in the knee, and it could offer a greater compatibility with tasks that are dominated by knee function or where an athlete needs to develop the knee moment as a ‘weak link’ [112, 113].

In contrast, some researchers have reported that the loaded jump squat exercise was more related to jumping and sprinting abilities than to the push press [87, 95]. This could be explained by the fact that Loturco et al. [87, 95] employed the push press instead of the push jerk, where the push jerk is known to be a faster exercise with unique proximal to distal recruitment strategy that is more related to improving jumping and sprinting performance [112–115]. Nonetheless, such a controversy shows the need to study WOPDs and their relationships to sporting tasks in greater depth, and consequently, also sport performance.

## Implementing Weightlifting Overhead Pressing Derivatives

The potential benefits of implementing WOPDs are not only to improve weightlifting performance, but also to enhance general sports performance, as discussed earlier. However, since many of the athletic population are not competitive weightlifters, they may not assume the same programming characteristics used by highly experienced weightlifters [88, 104, 108]. Consequently, adequate coaching and training strategies to implement WOPDs in a sports training programme remains to be determined.

The actual programming of WOPDs adapted to a sports program depends on the sport, the desired objective, and the time of the year that it is taking place [53, 54, 88, 104]. The benefits of weightlifting are best attained by strategically using the many weightlifting exercise variations according to their technical-complexity properties and speed-strength to strength-speed demands [23, 27, 53, 54, 65, 88, 104]. Empirical evidence indicates that implementing weightlifting movements and their derivatives may be a useful strategy to enhance sport performance, but the success may be negated if an incorrect technique is applied [23, 27, 53, 54, 88, 104]. Moreover, an adequate progression must be adopted in order to facilitate and reduce the time-consuming learning process in order to assure the benefits of implementing weightlifting exercises into a strength and conditioning program, and, finally, to reduce the risk of injury associated with a poor technique [44, 46, 54, 104, 116–118].

**Fig. 3:**
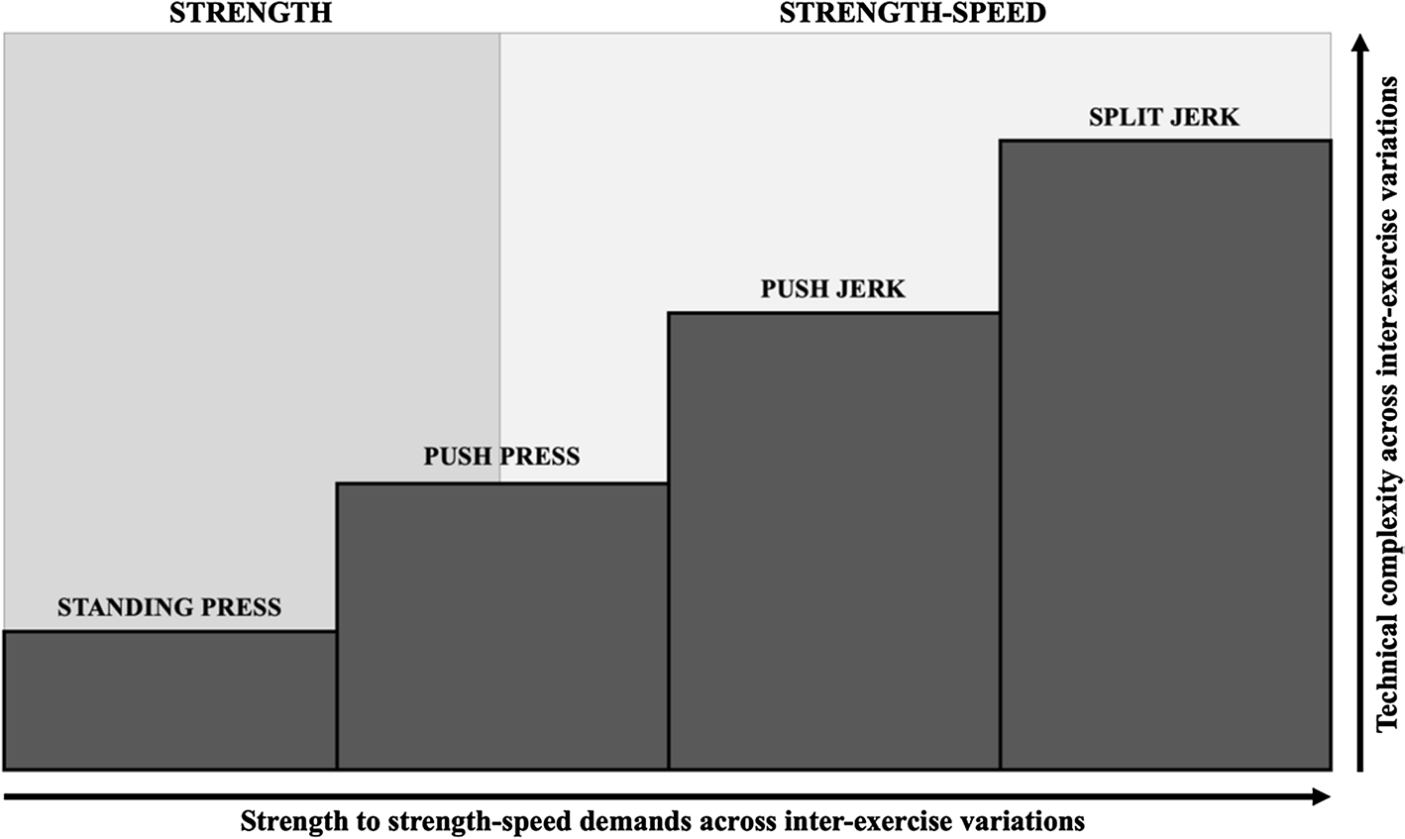
Graphic representation of a theoretical approach involving technical complexity, progression and strength-to-strength speed demands of WOPDs

Research on exercise technique reviews on WOPDs as well as weightlifting manuals suggest that a correct technical execution and progression is just as critical for success as choosing the right exercise [36, 45, 53, 54, 64, 68]. Based on this assumption a theoretical approach to cover the technical complexity, progression, as well as strength and power demands of WOPDs is illustrated in Fig. 3.

In summary, it may be useful to strategically use the performance parameters and progression according to technical complexity. Firstly, the usual process starts from the development of the slowest and least complex exercise, such as the standing press (non-ballistic nature) to form the basis of upper body strength, shoulder and thoracic complex mobility and overall motor control [53, 54, 64, 90, 103], to progress to more difficult and whole body strength-speed demanding exercises such as the push press, push jerk and split jerk (ballistic nature) [45, 53, 54, 68]. An adequate technical-complexity progression should follow that order.

Additionally, implementing WOPDs from the back is best learned for less experienced lifters facilitating the overall mechanical benefits, while avoiding a more technical complexity resulting from the weight being placed over the centre of gravity and thus requiring less torso strength to support the bar during the propulsion phase [36, 41, 43, 45, 51, 53, 54, 68, 71, 97]. It seems that implementing WOPDs from the back may be a useful strategy for the general sports population [45, 53, 54, 60, 68]; however, athletes who present with either a reduced shoulder flexion mobility or injuries in the shoulder complex should consider avoiding WOPDs from the back [119, 120].

Finally, weightlifters and more experienced lifters may implement more complex variations such as the squat jerk, snatch balance or snatch grip push press to target specific muscles and the snatch performance [44, 45, 51, 53, 54, 63, 101]. Specifically, weightlifters may choose to potentiate as a preferred exercise the push, squat or split jerk. The election of the jerk-style chosen will depend on individual characteristics of the lifters and the learning process that has been applied [41, 43, 45, 51, 60, 63]. Nonetheless, the split jerk remains the preferred exercise, and it is by far the most common exercise used in competitions.

## Conclusions and Practical Applications

To our knowledge, this is the first review of the literature on WOPDs that not only discusses weightlifting performance, but also provides information regarding historical, technical, kinetic and kinematics mechanisms, as well as potential benefits and guidelines for implementing the use of WOPDs as a potential method of training for the sporting population.

Practitioners may implement WOPDs such as the push press, push jerk or jerk from the back to provide an adequate stimulus to improve sport performance for several reasons. First, the use of WOPDs is a useful and well-supported strategy to improve weightlifting performance, due to the high number of failed attempts during the jerk phase in competition [97, 98, 105]. Second, WOPDs require the ability to develop force rapidly through the kinetic chain from the lower extremities to the upper extremities, which is mechanically similar to many sporting activities [55, 70, 111]. This movement pattern targets not only an impulsive triple extension of the ankles, knees and hips, but also optimizes motor control and coordination due to the key role of the trunk and lower body muscles in stabilising and transmitting forces in the closed kinetic chain [70, 90, 103, 106]. Third, WOPDs may be beneficial for enhancing power development and maximal strength in the sport population. This is supported by literature that has reported that WOPDs develop high levels of power output and allow for heavy loads to be lifted to an overhead position [48, 49, 88, 109].

Finally, the potential benefits reviewed in the literature with regard to WOPDs may be seen as clear enough reasons to implement them in sport training. However, relatively few investigations have been conducted to date. Only seven studies can be found from the last 20 years (see Fig. 2). Consequently, the contribution of this review is to establish a starting point, not only showing what has been developed in the literature to date, but also stating the need for future research.

## Limitations of the Current Study and Recommendations for Future Research

Based on the current literature and the information provided within this review, several potential limitations and research questions need to be addressed. The primary limitation is the limited research conducted to date and the poor progression registered in the last 20 years (see Fig. 2). Moreover, very few exercises, primarily push press and jerk (see Fig. 2), have been studied to date, which consequently leads to a lack of understanding regarding the kinetic and kinematic data on the full range of WOPDs. Furthermore, whilst the studies conducted on the push press used an amateur but well-trained population [78–80, 85, 87], the vast majority of studies that have analysed the jerk included in this review were conducted by highly trained professional lifters [16, 24, 26, 50, 72, 73, 86]. This may be one of the reasons why it is difficult to compare between studies, as highly experienced weightlifters have been shown to perform differently to their counterparts [50, 72, 73, 121]. Therefore, the kinematic and kinetic differences during WOPDs performed by different populations (gender, training status, etc.) certainly pose a research question that needs to be addressed.

Additionally, studies have shown a wide variability of the methodology of assessment during WOPDs. Some studies employed kinematic devices such as high-speed video cameras, LPT or accelerometers to assess barbell velocities and kinematic data (see Table 3) [16, 24, 26, 50, 86, 87]. In contrast, Comfort [80, 85] and Lake et al. [78] used force platforms to assess kinetic data and forces directly. Moreover, a few studies [72–74, 79] employed both force platform and high-speed video cameras to assess forces and velocities separately. Furthermore, whilst Lake et al. [78] and Flores et al. [16] selected a full range of loads to study the effect of load on the kinematic and kinetic variables, some studies employed a narrower range of loads [72, 73, 80, 87] or examined a single load [24, 26, 50, 74, 79, 80, 85, 86]. The differences in the methodology of assessment make it difficult to establish adequate comparisons between studies [122, 123]. Therefore, a standardized and well-defined assessment protocol used to identify the most adequate method to assess WOPDs remains unidentified in the literature.

Finally, barbell power and other kinematic variables have been studied during the jerk [50, 72, 73]. However, there is a lack of information regarding the longitudinal training effects that may result from implementing WOPDs, which should be researched in the future.
